# Predictors of Disease Recurrence Post Living Donor Liver Transplantation in End Stage Chronic HCV Patients

**DOI:** 10.1155/2014/202548

**Published:** 2014-02-18

**Authors:** Mostafa K. El Awady, Noha G. Bader El Din, Mahmoud Abdel Aziz Riad, Moataza H. Omran, Tawfeek H. Abdelhafez, Tamer Mahmoud Elbaz, Shereen Shoukry Hunter, Reham M. Dawood, Ashraf O. Abdel Aziz

**Affiliations:** ^1^Department of Microbial Biotechnology, National Research Center, Dokki, Cairo, 12622, Egypt; ^2^El Sahel Teaching Hospital, Ministry of Health, Al Kornich Shoubra, Cairo, Egypt; ^3^Department of Tropical Medicine, Kasr El Aini, Cairo University, Cairo, Egypt

## Abstract

HCV recurrence represents a universal phenomenon after liver transplantation. In this study Fifty HCV patients who underwent living donor liver transplantation were enrolled and factors that may accelerate HCV reinfection of the allograft such as donor's age and degree of liver steatosis, recipient's age, gender, BMI, MELD score, liver functions, HCV viral load, type of immunosuppressive drug, and genetic polymorphisms of IL28B, OAS, and IL1B were studied. The results of disease-free survival (DFS) rates showed inverse correlation with the recipient's postoperative levels of ALT, AST, ALP (*P* < 0.001, <0.001, and 0.006 resp.) as well as pre- and postoperative titers of HCV RNA (*P* < 0.003 and <0.001 resp.). Recipient's IL28B SNP was a significant factor in predicting postoperative DFS (*P* < 0.025). However, SNPs in OAS and IL1B genes had no apparent correlation with DFS. Cox proportional hazards model revealed that patients with elevated levels of ALT, preoperative viral titers, IL28B CT, and IL28B TT were 8.28, 4.22, 3.35, and 1.36 times, respectively, more likely to develop recurrence. In conclusion IL28B SNP, ALT level, and preoperative HCV titer besides proper choice of immunosuppressant are helpful for predicting posttransplant HCV recurrence and DFS.

## 1. Introduction

Chronic HCV infection is a leading cause of end-stage liver disease and hepatocellular carcinoma worldwide. Liver transplantation (LT) is a principal choice for treating patients with advanced liver disease. End stage liver disease associated with hepatitis C virus infection constitutes one of the principal indications for liver transplant. Unfortunately, reinfection of the graft (HCV recurrence) represents a universal phenomenon after LT and accounts for nearly half of all transplantations [1]. The majority of patients with virological recurrence will develop histological graft injury, chronic infection in 75–90% of transplant patients, and within 5 years, one-third of patients developing cirrhosis [2–4]. In addition, time interval from HCV cirrhosis to decompensation is significantly shorter in LT recipients than in nontransplant patients. It was reported that 5–30% of patients lose their graft due to HCV recurrence after liver transplantation [5, 6]. Also, HCV infection carried a higher risk of death 1 year after transplant as well as high risk of retransplantation. Generally, HCV recurrence decreased patient and graft survival in the long-term time frame [1].

The Egyptian population has a heavy burden of liver disease, mostly due to chronic infection with hepatitis C virus [7, 8]. The end stage liver disease (ESLD) secondary to HCV infection has become an increasing cause of mortality in Egypt in the last decades [9]. A study done by Yosry et al. [10] showed that hepatitis C related ESLD is the main indication for liver transplantation and represents 89.8% of cases in Egypt, while HBV and other indications (cryptogenic cirrhosis, Wilson disease, and glycogen storage disease) represent 5.1% and 5.1% respectively. Across different countries and transplantation programs, the 5-year posttransplantation survival rate for hepatitis C patients is significantly lower as compared to patients who underwent liver transplantation for other chronic liver diseases [11].

In the transplant setting, many factors contribute to disease progression compared with nontransplant patients. Factors associated with accelerated HCV recurrence after LT are related to the host (demographics, genetic background, immune status, sex, coinfections, and hepatic function at transplantation), the virus (genotype, viral replication, and viral quasispecies), and the donor (older age, degree of hepatic steatosis, weight, living versus cadaveric, anti-HCV status, and prolonged ischemic time), the environment (surgery-related factors, immunosuppression, alcohol). The viral and iatrogenic parameters were highly examined in many studies, while little is published on host factors [12]. Interestingly, in the immunocompetent population, the most powerful predictors of disease severity are those related to the host, while virus-related factors do not seem to play a major role in determining outcome. In particular, age at the time of infection, alcohol intake, and gender strongly affect disease progression [4, 13].

In addition, several factors have been discussed to influence the long-term outcome of graft such as the degree of human leukocyte antigen (HLA) matching or the IL28B genotype of the donor and the recipient [11]. There is now strong evidence for the important role of heritability in host immune responses to infectious pathogens and for susceptibility and outcome after exposure to microbial agents. Both the major histocompatibility complex (MHC) genes and non-MHC genes are increasingly identified as candidate genes with significant importance in the outcome of infectious diseases [12]. It was reported that any genetic variants that affect OAS1 activity could be important determinants of susceptibility/resistance to antiviral treatment and to virus-related disease progression [14]. Recently it was demonstrated that IL-28*β* SNPs play important roles in the management of hepatitis C virus (HCV) infections and are strongly associated with spontaneous and treatment-induced HCV clearance [11, 15, 16].

Identifying patients at risk for accelerated allograft loss is very important so that consideration can be taken in a timely fashion to use antiviral agents and/or decision to re-transplant. Therefore, there is a great need for biomarkers capable of accurately predicting HCV recurrence after LT and monitor disease progression. In the present study we will examine factors related to donor (age, liver steatosis), pre-transplant recipient (age, sex, HCV viral load, coinfections, SNPs of OAS gene, IL1*β* and IL-28*β* gene and posttransplant recipient immunosuppressive drugs, i.e., cyclosporine (CS), FK, and mycophenolate mofetil (MMF)), and their association with HCV recurrence following liver transplantation.

## 2. Subjects and Methods

### 2.1. Subjects

Fifty adult patients who underwent LDLT because of HCV induced end stage liver disease were enrolled and followed up in El Sahel Teaching Hospital. All the procedures were approved by the institution ethical review board according to Helsinki Declaration 1975 revised in 2008. Liver biopsies were taken at six and 12 months. Exclusion criteria include (coinfection with HBV, CMV or HIV, autoimmune hepatitis, alcoholic hepatitis, primary biliary cirrhosis, primary sclerosing cholangitis, fulminant hepatitis, and cryptogenic liver cirrhosis). Demographic data on recruited patients include detailed history, clinical examination, abdominal ultrasonography, liver biopsy, or fibroscan and laboratory testing including hematological and biochemical analyses. Evidence of HCV recurrence post liver transplantation depends on postoperative elevation of liver enzymes, hepatitis C viral replication demonstrated by polymerase chain reaction, and liver biopsy (histopathological diagnosis includes the presence of hepatitis activity index (HAI) score ≥6 and/or fibrosis staging ≥2 according to the Ishak modification of the Knodell classification). These are assisted six months after liver transplantation.

### 2.2. Blood Samples

Peripheral blood was withdrawn on EDTA. Genomic DNA was extracted using genomic DNA extraction kits (Qiagen, Milan, Italy). Sera were separated from coagulated blood for testing antischistosomiasis and autoimmune (ANA, AMA, and LKA) Abs. Hematological and biochemical tests (CBC, liver, and kidney functions) were analyzed for all subjects before and after transplantation. Since CMV reactivation was recently shown by our laboratory to interfere with several innate immunity pathways and subsequently poor rates of drug induced HCV clearance [17, 18], the presence of CMV DNA was explored in patients' genomic DNA after transplantation.

### 2.3. Detection of Exon 7 SAS Polymorphism of OAS 1 Gene

Genotyping for exon 7 splicing acceptor site (SAS) of OAS 1 gene (G/A) polymorphism was performed by a PCR-RFLP assay. A 203 bp PCR fragment was obtained following a protocol containing SAS specific primers as previously described [14]. Amplified product was digested with AluI (New England Biolabs, Hitchin, UK) and fragments were resolved by electrophoresis on 4% agarose gel containing ethidium bromide as described [14]. A band of 203 bp indicates the GG genotype, while bands of 150 bp + 53 bp indicate the AA genotype. The heterozygous genotype AG produces 3 bands of 203 bp + 150 bp  + 53 bp.

### 2.4. Genotyping of IL-1*β* + 3953 SNP

The region spanning the tested SNP was amplified by PCR using sequence specific primers; forward primer: 5′-GTTGTCATCAGACTTTGACC-3′ and reverse primer: 5′-TTCAGTTCATATGGACCAGA-3′. Reaction mix (50 *μ*L) contained 2 units Taq polymerase (Finnzyme, Finland), 5 *μ*L of 10X PCR buffer (supplied with the enzyme), 0.2 mM dNTPs (Promega, Madison WI, USA), 1.5 mM MgCl_2_, 5 *μ*M from each primer, and 3 *μ*L of DNA and DDW to 50 *μ*L. Thermal cycling protocol comprised initial denaturation at 96°C for 5 minutes, followed by 3 cycles each includes 96°C for 90 seconds, 53°C for 90, seconds and 72°C for 90 seconds, followed by 35 cycles each includes 96°C for 1 minute, 53°C for 1 minute, and 72°C for 1 minute and a final extension step at 72°C for 10 minutes. Amplified products were digested with 2 units Taq I restriction endonuclease (Amersham Pharmacia-Biotech, St Albans, UK) at 65°C for 3 hours. Restricted fragments were resolved on 2% agarose gel electrophoresis. A band of 249 bp indicates the TT genotype, while bands of 135 bp + 114 bp indicate the CC genotype. The heterozygous genotype CT produces 3 bands of 249 bp + 135 bp + 114 bp.

### 2.5. Detection of IL-28*β* rs12979860 C/T Polymorphism

Genotyping for the IL-28*β* rs12979860 C/T polymorphism was performed according to protocol described previously [15]. After BstU-I digestion the restricted products were resolved on 4% agarose gel electrophoresis containing ethidium bromide for staining. A band of 139 bp indicates the T/T genotype, two bands of 109 bp + 30 bp indicate the C/C genotype, and 3 bands of 139 bp + 109 bp + 30 bp (invisible) indicate the C/T heterozygous.

### 2.6. Statistical Analysis

The one-year disease-free survival (DFS) rates were correlated with various prognostic factors using Student *t*-test. All the prognostic factors that showed statistical significance <0.05 were introduced into Cox proportional hazards model for disease-free survival computation.

## 3. Results 

### 3.1. Clinical and Laboratory Findings of Patients Undergoing LDLT

The clinical data showed that 39 (78%) of patients were males, while 11 (22%) were females, with mean age of 49.9 ± 8.2 ranging between 17 and 60 years. The MELD score of the 50 patients before transplant ranged between 12 and 27 with a median of 18, while the BMI of patients ranged from 20 to 34 with median of 28. All the studied patients were Child C. The Child-Pugh score is used to assess the prognosis of chronic liver disease; mainly, cirrhosis and chronic liver disease are classified into Child-Pugh class A to C. Child-Pugh is now used to determine the prognosis, as well as the required strength of treatment and the necessity of liver transplantation. The laboratory data showed that all subjects were HCV RNA positive and negative for HBV, 26% of patients had HCC, and 54% had been exposed to schistosoma infection. The biochemical tests of all recipients are summarized in Table 1.

### 3.2. The Effect of Donor's Age and Liver Steatosis on HCV Recurrence

The donor age ranged from 21 to 40 years with the mean of 27.9 ± 9.2 years. Steatosis in the donor's liver ranged from 0% to 15% with a median of 3%. When comparing the donor's age and liver steatosis with the DFS we found that the donor's age (*P* = 0.235) and liver steatosis (*P* = 0.059) were nonsignificant factors affecting the postoperative disease-free survival as shown in Table 2.

### 3.3. The Effect of Recipient's Sex, Age, and BMI and MELD Score on HCV Recurrence

To study the role of the recipient's sex, age, BMI, MELD score, and presence of HCC on the DFS, all parameters were statistically compared with DFS rate. The results showed that 78% of recipient patients were males, while 22% were females, with a mean age of (49.9 ± 8.2) ranging between 17 and 60 years. The BMI of patients ranged from 20 to 34 with median of 28. The lowest MELD score of the 50 patients before transplantation was 12 and the highest was 27 with a median of 18 while all the studied patients were Child-Pugh C class. The correlation between DFS and recipient's sex, age, and BMI and MELD score is summarized in Table 3.

### 3.4. Correlation between Postoperative Laboratory Data and HCV Recurrence

Hematological and biochemical analyses including CBC, liver functions, kidney functions, alpha fetoprotein were tested for all subjects at the 6th and 12th month post-transplantation. Also, during the follow-up period samples were taken every six months. The relation of these tests with the postoperative DFS is summarized in Table 4. Postoperative ALT, AST, ALP, albumin, and total serum protein levels showed statistically significant changes with postoperative DFS rates, whereas other hematological and biochemical parameters had not significant correlation with DFS rates. The results shown in Table 4 indicated that only hepatic enzymes and liver specific proteins had statistical correlation with tendency of HCV recurrence. Changes in the remaining parameters seem to be not significantly correlated with DFS. Moreover, the minimal degree of hepatitis activity (A1) was shown in 75% of biopsies while (A2) was observed in the rest 25%. As for fibrosis, the majority 43.75% were (F1) while 31.25% were (F0) and the least percentage 25% was (F2). No advanced fibrosis (F3) or cirrhosis (F4) was detected in liver biopsies.

### 3.5. Association of HCV RNA Level, Bilharzial, and CMV Coinfection with HCV Recurrence

To examine the role of preoperative levels of HCV RNA, as well as earlier coinfection with schistosoma and CMV, DFS rate was correlated independently with each parameter. The results shown in Table 5 revealed that median levels of HCV RNA were (41 × 10^3^) and (62 × 10^3^) IU before and after transplantation, respectively. Both titers were significantly correlated with DFS rates. On the other hand neither preoperative schistosoma IgG titers nor CMV DNA correlated significantly with DFS. The current patient cohort includes 26% HCC with no apparent statistically significant correlation with DFS.

### 3.6. The Association of Recipients' Genetic Variants (OAS, IL1*β*, and IL28B Polymorphisms) with HCV Recurrence

To examine whether single nucleotide polymorphisms (SNPs) in some key genes play roles in determining the rate of HCV recurrence, SNPs of OAS, IL1*β*, and IL28B genes were studied in patients undergoing LDLT and were statistically correlated with rates of HCV recurrence. The results showed that the OAS and IL1*β* polymorphisms had no significant association with postoperative DFS. On the other hand the C/C genotype IL28B gene (rs12979860) displayed significant correlation with higher rates of postoperative DFS as shown in Table 6.

### 3.7. The Disease-Free Survival (DFS) in Relation to Immunosuppressive Drugs

The effects of three different immunosuppressive drugs on HCV recurrence were studied. The results shown in Table 7 showed that the use of MMF was significantly associated with higher rates of postoperative DFS (*P* < 0.004). On the other hand, the use of CS or FK had no significant effects on DFS rates after transplantation.

### 3.8. Cox Proportional Hazards Model for all Significant Factors

All factors showed significance level at 0.05 or less were entered into Cox proportional hazards model. Results showed that patients with abnormal ALT and high viral load before transplantation were more likely to develop recurrence than those with normal ALT levels and low viral load. As regards IL28B polymorphism, CC type was not associated with HCV recurrence, while patients with CT type and TT type were more likely to develop recurrence as shown in Table 8.

The results depicted in Table 8 denote that patients with abnormal ALT were 8.28 times more likely to develop recurrence than those with normal ALT levels. Elevated viral RNA titers before transplantation were found to be a significant factor as patients with higher viral load before transplantation were 4.22 times more likely to develop recurrence (*P* value = 0.028). Regarding IL28B polymorphism, CC genotype was clearly protective against HCV recurrence, while patients with CT genotype were 3.35 times more likely to develop recurrence and patients with TT genotype were 1.36 times more likely to develop recurrence though not statistically significant.

## 4. Discussion

Chronic infection with HCV is a leading indication for liver transplantation [19]. The poor outcome of HCV-positive recipients has resulted in divergence in the transplant outcomes between HCV-positive recipients and HCV-negative recipients. Improvements in organ preservation, surgical techniques, and postoperative care have dramatically improved the survival of HCV-negative recipients over the last two decades, whereas this has not been the case in HCV-positive recipients for whom outcome has remained unchanged or even worsened over time [20]. There is a great demand for identifying the factors related to severe hepatitis C recurrence and disease progression. This study was carried out on 50 patients with chronic HCV-related liver disease who underwent living donor liver transplantation. The current results showed that donor's age did not seem to affect the rate of HCV recurrence since the majority of donors in this study were under 40 years old with a mean age of 27.9 ± 4.7 years, thus supporting the findings of Alonso et al., Lake et al., and Rayhill et al. [21–23] showing that donor's age is the primary risk factor for recurrent hepatitis C with increased severity of recurrence at donors' ages >40 years. Degree of steatosis in donors' livers did not exceed 15% consequently avoiding the possibility of having worse outcome when receiving grafts with > 30% steatosis as reported by Briceño et al. [24]. Regarding recipient's sex, Lai et al. [25] reported that female sex was an independent predictor of advanced recurrent disease and mortality, a finding that could not be supported by our data showing that sex of recipient does not affect the postoperative disease-free survival (*P* = 0.776). Belli et al. [26] reported a synergistic effect between donors' age and recipients' female sex, which highlights the importance of using younger donors for female recipients. Liver function tests were repeatedly shown to significantly affect posttransplantation recurrence [27, 28]. Specifically, our data showed that elevated ALT, AST, and ALP levels were associated with increased risk of HCV recurrence (*P* < 0.001, <0.001 and <0.006 resp.) after LDLT. However, changes in serum levels of GGT did not correlate significantly with DFS rates neither in the current study (*P* = 0.915) nor in the results reported by Feurer et al. [27]. The currently observed data on serum bilirubin and INR posttransplantation (*P* = 0.709 and 0.587 resp.) did not reproduce the results of Charlton et al. [28] who showed that bilirubin and INR are predictors of posttransplant mortality and suggested that these 2 variables reflect general debility of the recipient.

Ghabril et al. [29] found that high viral load in addition to high grade of fibrosis and older donor age is predictors of advanced fibrosis. Our results showed that Hepatitis C viral load before and after transplant (3–6 months) is highly significant factors affecting HCV recurrence after liver transplantation (*P* < 0.003 and *P* < 0.001 resp.). In this context, the current results support earlier reports of Khettry et al. [30] and Ciccorossi et al. [31] that high viral load pre-LT was correlated with more progressive recurrence of HCV and higher early (1 week) posttransplantation viral loads (≥2.5 × 10^5^ IU/mL) are at risk for significant recurrent hepatitis C. These data suggest that patients who are at risk for significant HCV recurrence can be identified early.

Although cytomegalovirus (CMV) infection was thought to be associated with more progressive chronic HCV disease and worse recurrent HCV, possibly via its association with host immunosuppression, the current data showed no association of CMV infection of the recipient with disease-free survival (*P* ≤ 0.801), thus supporting earlier reports who failed to show a correlation between CMV infection and HCV recurrence [32, 33]. Burak et al. [34], on the other hand, reported that graft failure was significantly more common in patients with CMV infection versus those without CMV.

We currently examined the association of 2′, 5′-oligoadenylate synthetase (OAS), interleukin 1*β* (IL1*β*), and Interleukin 28*β* (IL28B) polymorphisms with the rate of HCV recurrence following liver transplantation. Results showed that the heterozygous form of OAS A/G was more common than A/A and G/G with highest rate of 6 months disease-free survival (51.3% for A/G versus 25.0 and 0.0% for A/A and G/G resp.) but was statistically nonsignificant (*P* ≤ 0.133). These data were contradictory to those published by Whitehill, [35] who reported that OAS genotype A/G had the highest rate of HCV recurrence post-transplantation within 12 months. Although IL1*β* has been shown to be a key cytokine in development of steatosis and hence liver fibrosis, the current data showed no significant association of IL1*β* polymorphism with HCV recurrence after transplantation (*P* ≤ 0.425). To the limit of our knowledge, no published data are available on the association of IL1*β* SNP with DFS rates after LDLT. However, IL1*β* SNPs were found to affect the outcome of hematopoietic stem cell transplantation as it was one of gene variants which have been reproducibly associated with graft-versus-host disease (GVHD) [36, 37]. In contrary, genetic variations of IL28B had statistically significant association with the severity of HCV recurrence after LDLT. Recipients with genotypes (CT and TT) had higher risk of severe HCV recurrence than those bearing a CC genotype (*P* ≤ 0.025). These results agree well with the previous reports on associations of IL28B SNP with histologic recurrence of HCV posttransplantation [38, 39]. We can then speculate that genetic variants in IL28B gene might be associated with histological recurrence of HCV and are reproducible predictors of complications during the first year after LT.

A tacrolimus based regimen (with adjusted dose according to patient's trough serum level) is the main immunosuppression protocol used in this study. This regimen is accompanied with steroids that taper gradually till completely withdrawn at the end of the twelfth week. Shift of tacrolimus to cyclosporine occurs in patients who experience tacrolimus related side effects. Recently, Kim et al. [40] compared recipients of LDLT who received cyclosporine and tacrolimus as immunosuppressive agents. Their results indicated that graft and patient survival rates were similar in the 2 groups, but histologic HCV-recurrence-free survival was significantly higher in the cyclosporine group. Also, Irish et al. [41] found that patient and graft survival rates with cyclosporine and tacrolimus were relatively similar. Thus, there was no definitive evidence in favor of one immunosuppressive agent over the other for reducing the risk of recurrent hepatitis C, and selection of immunosuppressive agents should be based on individual patient's data. We also observed that the use of either immunosuppressant had no statistical correlation with DFS rates (*P* ≤ 0.33 and *P* ≤ 0.23 for CS and FK resp.). In this study, the addition of mycophenolate mofetil (MMF) significantly increases HCV recurrence and severity (*P* ≤ 0.004), thus supporting the results of Nelson et al. [42], who reported severe hepatitis C recurrence in patients subjected to induction with MMF when compared with standard therapy based on tacrolimus. Klintmalm et al. [43] reported that the overall intensity of immunosuppression rather than the independent action of either drug may have greater impact on HCV recurrence. On the other hand, Jain et al. [44] compared tacrolimus, mycophenolate mofetil, and prednisone to tacrolimus and prednisone alone and found that there were no differences in graft loss or rate of HCV recurrence. In our attempt to benefit from the designed Cox proportional hazards model we found that patients with abnormal ALT and high viral load before transplantation were 8.28 and 4.22 times, respectively, more likely to develop recurrence while patients with IL28B genotype CT and TT were 3.35 and 1.36 times, respectively, more likely to develop recurrence. In conclusion, assessment of IL28B polymorphism, level of ALT, HCV RNA load before transplantation, and proper choice of immunosuppressant agents are helpful predictors for posttransplantation recurrence and disease severity. Future studies on larger cohorts are required to evaluate potential roles of different genetic polymorphisms and to plan for proper management strategies.

## Figures and Tables

**Table 1:** Laboratory data of patients undergoing LDLT. Blood samples were withdrawn just before surgery. Sera were separated and analyzed for the above parameters.

	Mean ± SD	Medium	Minimum	Maximum
ALT	67.5 ± 64.4	47.5	6.0	290.0
AST	63.3 ± 56.5	44.5	7.0	206.0
ALP	210 ± 172	159.5	40.0	902.0
GGT	407 ± 1270	104.5	20.0	9048.0
T. Protein	7.1 ± 0.8	7.1	5.1	8.9
Albumin	4.1 ± 0.5	4.2	2.8	5.0
T. Bilirubin	1.3 ± 1.0	1.0	0.3	4.3
D. Bilirubin	0.5 ± 0.5	0.3	0.1	2.3
PT	13.7 ± 1.1	13.4	12.0	17.0
PC	89.9 ± 14.5	97.5	45.0	100.0
INR	1.1 ± 0.2	1.0	0.9	1.9
Creatinin	1.0 ± 0.5	1.0	0.4	3.0
K	4.4 ± 0.6	4.4	3.5	6.7
Na	138.3 ± 4.1	138.0	128.0	146.0

**Table 2:** Correlation between DFS and donor's age and liver steatosis. Patients undergoing LDLT were divided into 2 groups according to age (below and above 25 y) and according to degree of liver steatosis (below and above 3%). DFS rates were calculated during the first 6 months after surgery and correlated with age and steatosis groups.

	No. of cases	12 m DFS	*P* value	Sig.
		(%)		
All	50	20.6		
Age				
<25	20	36.5	0.235	NS
>25	30	21.5		
Steatosis (%)				
<3	27	51.6	0.059	NS
>3	23	0.0		

**Table 3:** Correlation between DFS and recipient's sex, age, BMI, MELD score, and HCC. Host parameters including sex ratio, age of participants, and BMI and MELD score were determined and were statistically analyzed. The results showed that all host parameters were nonsignificant factors affecting the post operative disease-free survival (*P* value = 0.776, 0.064, 0.466, and 0.663, resp.).

	No. of cases	12 m DFS	*P* value	Sig.
		(%)		
All	50	20.6		
Sex				
Females	11	0.0	0.776	NS
Males	39	25.9		
Age				
<50	23	31.4	0.064	NS
>50	27	27.2		
BMI				
<25	7	50.0	0.466	NS
>25	43	21.3		
Meld score				
<18	30	23.3	0.663	NS
>18	20	43.5		
HCC				
+	13	33.8	0.172	NS
−	37	23.1		

**Table 4:** Correlation of postoperative hematological and biochemical parameters with DFS. Several key biochemical and hematological parameters were assayed at the 6th month after LDLT surgery and were correlated with the computed DFS rates that reflect HCV recurrence.

	No. of cases	12 m DFS	*P* value	Sig.
		(%)		
All	50	20.6		
Hb.				
<11	11	85.7	0.136	NS
>11	39	0.0		
WBCs				
<4000	9	25.0	0.052	NS
4000–11000	21	23.9		
Platelets				
<150000	20	23.7	0.663	NS
>150000	30	30.8		
ALT				
<37	28	78.1	<0.001	**HS**
>37	22	0.0		
AST				
<41	26	100.0	<0.001	**HS**
>41	24	0.0		
ALP				
<160	26	34.9	0.006	**HS**
>160	24	0.0		
GGT				
<105	25	53.9	0.915	NS
>105	25	19.2		
T. proteins				
<7	25	12.7	0.027	**S**
>7	25	74.2		
Albumin				
<4	17	9.8	0.017	**S**
>4	33	71.8		
T. bilirubin				
<1.0	35	0.0	0.709	NS
>1.0	15	36.6		
D. bilirubin				
<0.3	30	58.8	0.179	NS
>0.3	19	15.9		
PT				

<13.5	25	0.0	0.615	NS
>13.5	25	27.5		
PC				
<97.5	25	19.9	0.869	NS
>97.5	25	46.7		
INR				
<1	26	0.0	0.587	NS
>1	24	26.5		
Urea				
<25	25	23.9	0.522	NS
>25	25	0.0		
Creatinine				
<1.2	45	39.7	0.596	NS
>1.2	5	0.0		
*α* fetoprotein				
<3.3	25	0.0	0.942	NS
>3.3	25	47.9		

NS: nonsignificant statistical correlation, S: significant, and HS: highly significant.

**Table 5:** Correlation of HCV RNA titers, schistosoma IgG, and CMV DNA with DFS. Each of the abovementioned parameters was divided into 2 groups based on the HCV titer whether above or below the calculated median, presence or absence of IgG Ab, or presence or absence of CMV DNA were statistically compared with DFS rate.

	No. of cases	12 m DFS	*P* value	Sig.
		(%)		
All	50	20.6		
HCV PCR Before Transpl.				
<43000	25	82.9	0.003	**HS**
>43000	25	0.0		
HCV PCR Post Transpl.				
<62500	25	100.0	<0.001	**HS**
>62500	25	0.0		
Bilharzial Ab				
+ve	27	51.6	0.059	NS
−ve	23	0.0		
CMV PCR Post-Transpl.				
−ve	30	0.0	0.801	NS
+ve	20	22.5		

**Table 6:** Correlation of single nucleotide polymorphisms in the OAS, IL1B, and IL28B genes with DFS rates. Total genomic DNA was extracted from patients undergoing LDLT and typed for specific SNPs in or around OAS, IL1B, and IL28B genes. The distribution of different variants was statistically correlated with DFS rates after transplantation.

	No. of cases	12 m DFS	*P* value	Sig.
		(%)		
All	50	20.6		
OAS polymorphism				
AA	5	25.0	0.133	NS
AG	40	51.3		
GG	5	0.0		
IL1B polymorphism				
CC	22	0.0	0.425	NS
CT	17	30.5		
TT	11	18.2		
IL28B polymorphism (rs12979860)				
CC	10	62.5	0.025	**S**
CT	31	0.0		
TT	9	0.0		

**Table 7:** Correlation of immunosuppressive agents and DFS posttransplantation. The results show that the use of MMF was highly significant factor affecting the postoperative disease-free survival (*P* value = 0.004), while the use of CS and FK was nonsignificant (*P* value = 0.337 and 0.231 resp.).

	No. of cases	12 m DFS	*P* value	Sig.
		(%)		
All	50	20.6		
CS				
C.S	**15**	0.0	0.337	NS
None	**35**	21.3		
FK				
F.K	**33**	20.6	0.231	NS
None	**17**	0.0		
MMF				
MMF	**32**	0.0	0.004	**HS**
None	**18**	66.7		

**Table 8:** Cox proportional hazards model for all significant factors. The most important 3 factors affecting the HCV recurrence after LDLT were subjected to Cox proportional hazards analysis where B is the regression coefficient, SE is the standard error or means, and HR is the hazardous ratio.

	B	SE	*P*-value	HR	95.0% CI for HR	
					Lower	Upper
Postoperative ALT	2.125	0.648	**0.001**	8.28	2.35	29.82
Preoperative HCV titer	1.439	0.656	**0.028**	4.22	1.17	15.24
IL28B						
CC	Reference		0.223			
CT	1.210	0.863	0.161	3.35	0.62	18.19
TT	0.310	0.805	0.700	1.36	0.28	6.61

*P* value < 0.05 is considered significant.
